# Continuous High‐Throughput Plasma Separation for Blood Biomarker Sensing Using a Hydrodynamic Microfluidic Device

**DOI:** 10.1002/adhm.202404193

**Published:** 2025-02-19

**Authors:** Hesam Abouali, Fatemeh Keyvani, Seied Ali Hosseini, Sanjana Srikant, Mahla Poudineh

**Affiliations:** ^1^ Department of Electrical and Computer Engineering University of Waterloo Waterloo ON N2L 3G1 Canada

**Keywords:** automated analysis, aptamer biosensor, biomarker, blood plasma separation, microfluidic

## Abstract

Continuous, cost‐effective, high‐throughput with admissible yield and purity of blood plasma separation is widely needed for biomarker detection in the clinic. The existing gold standard technique (centrifugation) and microfluidic technologies fall short of meeting these criteria. In this study, a microfluidic device design is demonstrated based on passive hydrodynamic principles to achieve admissible yield and purity plasma samples. Through computational and experimental assessments, it is shown that side channels with varying lengths are required to improve the plasma extraction rate. The optimized side channels in this device design use the formed cell‐free layer regions in the expanded areas to extract plasma consistently and efficiently. These Hydrodynamic Continuous, High‐Throughput Plasma Separator (HCHPS) microfluidic devices achieve a purity in the range of 47% to 64% with whole blood and maintaining a yield of 10% to 18%, with half hemolysis compared to gold standard centrifugation. These devices also separate the plasma from diluted blood with a purity in the range of 62% to 97% with a similar yield range. Additionally, whole human blood spiked with lactate was processed through the HCHPS device, and the separated plasma is collected and analyzed using two biosensing approaches, a bead‐based fluorescence, and an electrochemical aptamer biosensing, confirming the quality of plasma for downstream biomarker detection.

## Graphical abstract

Plasma separation from whole blood is a necessary part of biomarker detection. Using conventional centrifugation undermines the goals of point‐of‐care devices and more automated biomarker analysis workflow. In this work, a high‐throughput hydrodynamic‐based microfluidic platform that separates plasma with admissible purity and yield is developed, facilitating automated biomarker detection.

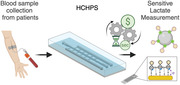

## 1 Introduction

Blood plasma is the most abundant source of biomarkers, such as proteins, metabolites, peptides, nucleic acids, cytokines, and hormones. These biomarkers have been scrutinized in neurological disorders like multiple sclerosis^[^ ^1^, ^2^ ^]^ and Alzheimer's disease,^[^ ^3^, ^4^ ^]^ in precision oncology,^[^ ^5^, ^6^ ^]^ and in diabetes mellitus.^[^ ^7^, ^8^ ^]^ Their detection is crucial for disease diagnosis and monitoring treatment progress. In conventional biomarker analysis, separating biomarker‐containing plasma from other blood components is an essential initial step and it is usually conducted through manual centrifugation. However, the limitations of this process can be restrictive. Although centrifugation approaches offer high yield and purity compared to passive and hydrodynamic‐ based plasma separation,^[^ ^9^, ^10^ ^]^ centrifugation is time‐consuming, needs expensive and bulky equipment, and is limited in sample throughput. Besides, it cannot be integrated with downstream analysis.^[^ ^11^, ^12^, ^13^ ^]^

Consequently, it is necessary to tackle all these concerns, specifically addressing the constraints in high‐throughput and continuous plasma separation to prepare high‐quality samples for downstream analysis. For this purpose, various techniques have been integrated into microfluidics for plasma separation, including active methods like acoustofluidics and dielectrophoresis, which depend on the implantation of external forces in the microfluidic channels, or passive methods such as membrane‐based filtration, inertial separation, and hydrodynamically dependent geometries, which do not require any external force.^[^ ^9^, ^14^, ^15^ ^]^

There is a preference for hydrodynamic‐based passive devices over active devices primarily due to their cost‐effectiveness, simplicity, as well as their independence from external force fields for operation.^[^ ^13^ ^]^ Additionally, among the passive methods, hydrodynamic‐based systems ensure continuous, high‐throughput, and clog‐free operation, making them more advantageous than other passive methods like filtration or sedimentation.^[^ ^13^ ^]^

By leveraging a combination of varying hydrodynamic forces and effects, cells, particularly deformable cells such as red blood cells (RBCs) or white blood cells (WBCs), can be focused, and blood plasma can be separated. In a prior study,^[^ ^16^ ^]^ plasma was obtained through a side channel branching off from a contraction‐expansion area (CEA) where a cell‐free layer (CFL) is formed. The CFL formation occurs due to the Fåhuaes–Lindqvist effect, where cells tend to move toward the centreline of a microcapillary vessel, leading to their concentrating in the lateral position.^[^ ^17^ ^]^ This CFL is formed in a CEA when dominant hydrodynamic forces like inertial lift and viscous drag forces are in equilibrium at low Reynolds numbers.

Subsequently, the Zweifach‐Fung bifurcation effect can be used to extract plasma from branched side channels.^[^ ^18^ ^]^ To employ these hydrodynamic effects for plasma separation, the conventional bifurcation methods utilize narrow channels with a width comparable to the cell diameter that is located on both sides of a main channel where blood enters. The hydrodynamic resistance of such constricted side channels is considerably high, and as a result, a very limited portion of the blood plasma is isolated, leading to a low extraction yield of 5%^[^ ^16^ ^]^ and loss of important, rare biomarkers. Despite this, while working with undiluted samples and at typical hematocrit levels (range of 40–45%), there is a trade‐off between purity and yield, resulting in a compromise of one of these factors in the device.

**Figure 1:**
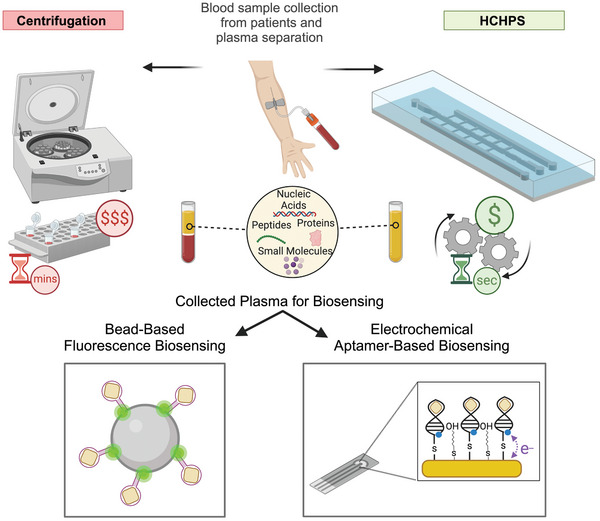
Overview of blood plasma separation. The plasma separation with HCHPS can be performed continuously and with high‐throughput without a need for bulky and expensive equipment such as a centrifuge in a shorter time. However, the separation of plasma conventionally with the centrifuge needs access to this equipment, which can be time‐consuming, in batch mode, and unable to provide continuous plasma samples for downstream analysis. To demonstrate this and assess the quality of extracted plasma, plasma collected from the HCHPS device was used for lactate molecule detection through two different biosensing approaches. Created with BioRender.com.

Here, we report a new Hydrodynamic Continuous, High‐throughput Plasma Separator (HCHPS) microfluidic device, to enhance the previously developed design^[^ ^16^ ^]^ for admissible ‐yield and ‐purity plasma separation (**Figure** **1**). In our new design, we showed that side channels with different lengths created varying hydrodynamic resistance, which lead to an increase in the extraction yield while keeping the purity of extracted plasma at acceptable levels. We designed three new devices that can achieve a plasma extraction with a maximum purity of 63.81% and a maximum yield of 35% working at high throughputs of 5 to 15 mlh^−1^ while separating plasma from whole blood samples. Also, in case of operation with diluted blood samples, a maximum purity of 96% and a yield of 35% were achieved. Moreover, the quality of the extracted plasma is better than centrifugation since hemolysis occurs less in the microfluidic device (54% decrease).

The HCHPS enables continuous processing of blood samples at a high‐throughput, separates plasma with acceptable purity, yield and quality. Although this device has less purity and yield compared to gold‐standard centrifugation, the ease of use and accessibility of this device compared to a centrifuge make it more feasible for downstream analysis, such as biomarker detection in a sensitive way using an aptamer bead‐based fluorescence biosensing and an electrochemical aptamer‐based biosensor (EAB). The HCHPS has the potential to move us toward an automated patient sample processing and analysis and can be easily integrated with downstream modules for different biomedical applications including the biosensing of blood biomarkers.

## 2 Results and Discussion

### 2.1 Design Concept and CFD Studies

**Figure 2:**
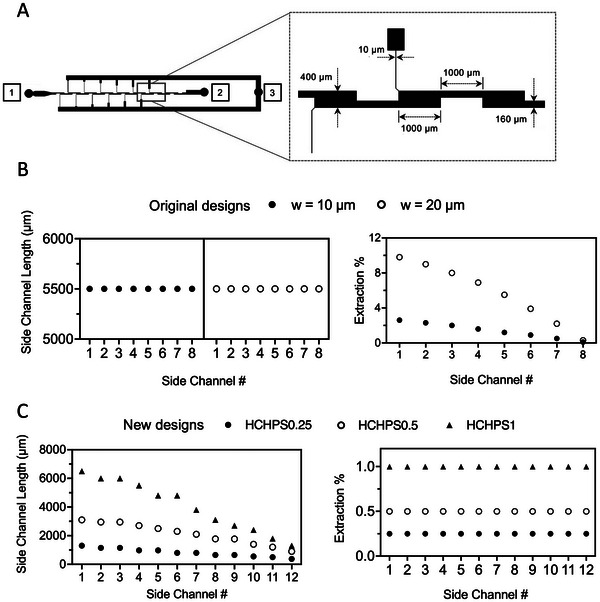
Design of the HCHPS device and CFD simulation results. A) The general layout and the dimensions of the HCHPS devices are shown. The blood enters the device from port #1, and waste, which contains RBCs/WBCs exits the device from port #2; the extracted plasma is collected from port #3. B) Shows the simulation results from the original device. This graph suggests that the last four side channels have limited drainage capability, which negatively affects the yield from the devices. C) The simulation for the new designs has been conducted to calculate the required length to avoid such a problem in this work. The simulation results show that a good yield can be achieved while maintaining an admissible purity. Each data point shows the result from a single simulation. The simulation results were consistent for multiple runs, thus only a single run is shown in the graphs.

The newly developed HCHPS microfluidic devices were designed following a previous study^[^ ^16^ ^]^ (original devices), These HCHPS microfluidic devices contain a main channel with widths of 400 µm and 160 µm in expanded and contracted parts, respectively, and a height of 50 µm. The main channels consist of sixteen CEAs, where CFL forms. Asymmetrical parallel side channels were placed at the corner of the first twelve CEAs for plasma drainage. In contrast to the side channels in the original devices, which have varying 10 µm and 20 µm width with a constant length, the side channels in the new HCHPS devices differed in length. This variation was designed to compensate for the low yield of the original work. The side channels at each side of the main channel are linked to two plasma collecting channels. All side channels have a width of 10 µm and a height of 20 µm. **Figure** **2**A shows all the parts and their dimensions.

To maximize yield and maintain admissible‐purity levels, we employed 3D computational fluid dynamics (CFD) to determine the optimal side channel length and flow rate. To achieve a higher yield, our goal was to keep the extraction rate, which is indicated by the flow rate ratio (FRR), constant throughout the device. And, to achieve a high purity, we investigated different operational flow rates. We also conducted a CFD study for both original devices. As shown in Figure 2B, all the side channels in the original device had a length of 5.5 mm. The CFD results showed that for the original 20 µm design, the first side channel extracted 9.8% of the downstream flow, the second side channel extracted 9.0%, and the third one drained 8%. However, the last three side channels could extract 3.9%, 2.2%, and 0.3% respectively. While this device showed a satisfactory plasma extraction yield of 30%, the purity suffers due to the 20 µm side channel width and reaches 32% with a hematocrit (HCT) level of 33.7% (diluted blood) with a throughput of 10 mlh^−1^. The trade‐off between the purity and the yield could be seen for the 10 µm device. In this device, the first three side channels could extract 2.6%, 2.3%, and 2.0%. On the other hand, the extraction rate for the last three side channels dropped significantly to only 0.9%, 0.5%, and 0.1%, which means that those final side channels could not extract as efficiently which was confirmed in experiments with whole blood samples. Although the purity for this design was 99% for whole blood (HCT 45%), the yield for this design was 5% and the throughput was 2 mlh^−1^.^[^ ^16^ ^]^

With the new designs, the extraction rates for all side channels had been set to 0.25%, 0.5%, or 1% in HCHPS devices. The devices are called HCHPS1 (1% extraction rate), HCHPS0.5 (0.5% extraction rate), and HCHPS0.25 (0.25% extraction rate.) With this change in the design, all side channels could maintain a constant extraction rate from the downstream flows, resulting in an increase in the yield of the extracted plasma. As shown in Figure 2C, the side channel length for the design with the extraction rate of HCHPS1, had longer side channels, compared to the other two HCHPS devices.

### 2.2 Cell‐Free Layer Formation

**Figure 3:**
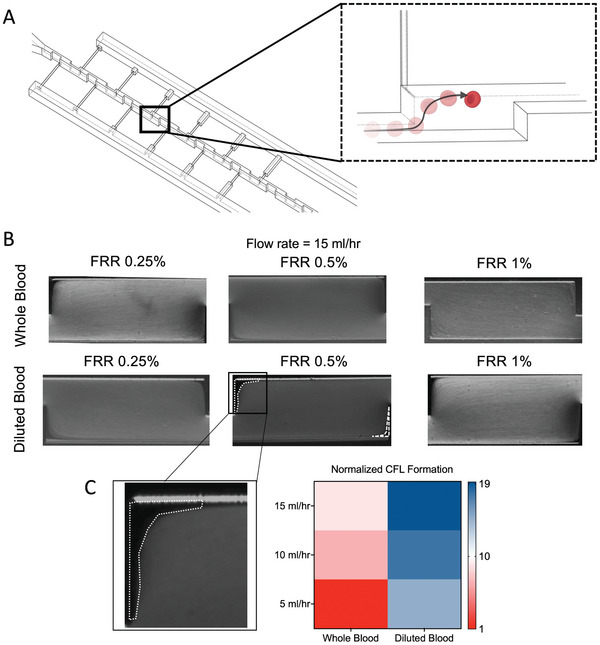
CFL formation evaluation. A) illustrates how cells pass through the CEA forming the CFL. B) images of the formed CFL under the microscope with whole and 1:1 diluted human blood samples at a flow rate of 15 mlh^−1^ for the HCHPS0.5 design. C) CFL was imaged and its area ratio (*F*/*F*_*min*_; CFL area (*F*) normalized to CFL area with whole blood at 5 mlh^−1^ (*F*_*min*_)) was calculated for HCHPS0.5 at different flow rates with human whole blood and 1:1 diluted blood. The heatmap results show one experimental replicate. With an increase in the flow rate up to 15 mlh^−1^, we could achieve a suitable CFL area that made a better quality plasma separation possible.

The plasma separation within the HCHPS devices relies on the formation of the CFL at the corners of each CEA, as previously discussed. The schematic illustration (not to scale) in **Figure** **3**A shows the flow of RBCs passing from the constriction area to the expansion area. With a centreline‐focused stream of RBCs/WBCs, the CFL forms. This CFL formation heavily depends on the blood hematocrit level, CEA dimensions, and the operational flow rate.

Experiments were conducted with whole blood and diluted blood (HCT level 22.5%) to evaluate the performance of the new devices in forming CFL. First, the human whole blood was run through all three new devices at different flow rates, starting from 5 to 10 mlh^−1^, and 15 mlh^−1^. As Figure 3B demonstrates, the HCHPS0.25 design showed a larger formed CFL compared to HCHPS0.5 and HCHPS1. The results were supported by previous study,^[^ ^19^ ^]^ indicating that as extraction yield increases, there is a compromise in the purity of the collected plasma. The new HCHPS designs address this particular issue, and, clearly, HCHPS0.5 achieved a better CFL compared to HCHPS1. CFLs for flow rates of 5 and 10 mlh^−1^ can be found in the Supporting Information, as shown in Figure S1 (Supporting Information). To visually present this formed CFL, images of the formed CFLs are shown for diluted blood as well (Figure 3B). The dilution of blood prior to analysis is not preferred as it dilutes the concentration of analytes of interest and requires a more sensitive biosensor to be developed for its detection and quantification. Given that the HCHPS0.5 device optimally balances yield and purity, we calculated its CFL area under various conditions. The normalized CFL area indicates that 15 mlh^−1^ for whole blood can be an ideal operation flow rate (Figure 3C).

### 2.3 Device Performance Characterization

The newly designed devices (HCHPS0.25, HCHPS0.5, and HCHPS1) were evaluated for their plasma separation capability. The efficiency of a plasma separation device is determined by three main criteria, yield, purity, and quality. The extracted plasma's purity reflects the quantity of unwanted blood cells in the extracted plasma. This analysis is done with the help of flow cytometry to measure the concentration of cells at each outlet and inlet (sample data shown in the Figure S2, Supporting Information). The purity level was determined by the decrease in the concentration of blood cells discharged from the plasma outlets compared to the total cells concentration entering through the inlet (Equation (2)). The second key factor is the yield of the device, which indicates its effectiveness in extracting plasma from whole blood. The yield was calculated by measuring the volume of both outlets (Equation (3)). Finally, the quality of the blood was determined by the degree of hemolysis taking place inside the device due to hydrodynamic and physical forces.

**Figure 4:**
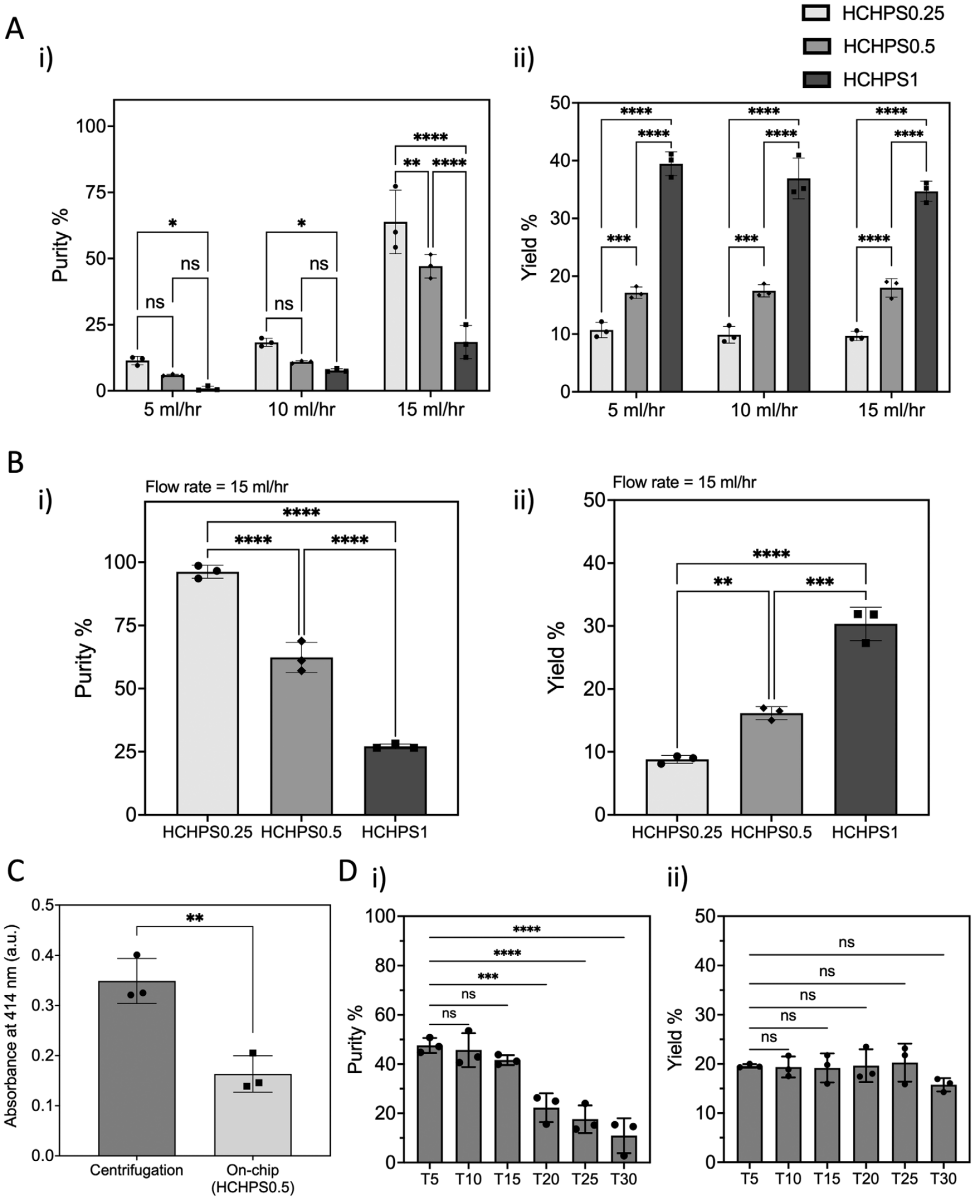
HCHPS performance. A) shows the performance of HCHPS devices at different flow rates with whole blood. Among these flow rates, 15 mlh^−1^ shows a better purity (i), however, the yield (ii) is not impacted by the flow rate rather it depends on the lengths of the side channels and the extraction rate. B) The same evaluations have been done for diluted blood. For the diluted blood, the purity (i) reaches 96.25% for HCHPS0.25 and 62.32% for HCHPS0.5, and 27.08% for HCHPS1 devices. The yields (ii) are very similar to the whole blood samples as the HCT level does not affect the yield. C) the quality of plasma was assessed for separation via centrifugation and on‐chip via HCHPS0.5 device, based on the level of hemolysis. The results show that on‐chip extraction produces a higher‐quality plasma since the erythrocytes remain intact. D) The performance of the HCHPS0.5 device was evaluated for continuous plasma separation (30 min working time). The results suggest that the device can reliably work for 15 min, maintaining its purity (i) and yield (ii). The data shows the mean ± standard deviation of three replicates. A) and B) the comparisons between groups are done with two‐way ANOVA with Tukey's multiple comparisons test: 0.1234(ns), 0.0021(**), 0.0002(***), < 0.0001(****). C) The comparisons between groups are done with an unpaired t‐test with Welch's correction. p values: 0.001 to 0.01 (**). D) The comparisons have been done by ordinary one‐way ANOVA. p values: 0.1234 (ns), 0.0332 (*), 0.0021 (**), 0.0002 (***), <0.0001 (****).

**Figure** **4**A‐i,ii show the purity and yield of plasma extracted by HCHPS devices from human whole blood at different flow rates. The same criteria were evaluated for diluted blood and are shown in Figure 4B. The quality of the separated plasma is also shown in Figure 4C. The HCHPS1 device, as expected, showed a better yield compared to other designs. With increasing the flow rate of the sample, the CFL area increased as cells were placed closer to the channel centreline. The HCHPS0.5 device showed a purity of about 47.08% while the HCHPS0.25 design has a purity of 63.81%. Moreover, HCHPS0.5 device yielded a 17.93% plasma compared to 9.69% of the HCHPS0.25 device. Although the yield of the HCHPS1 is higher than other designs, about 34.68%, its purity is lower at 18.44%. All designs were evaluated with the diluted blood sample (1:1 diluted) at the highest flow rate (15 mlh^−1^) for both performance criteria (Figure 4B). To conclude the device selection for further utilization, HCHPS0.5 was chosen since it provides a better yield compared to HCHPS0.25 (significant difference) and achieved a significantly better purity compared to HCHPS1.

In the pre‐processing step of the blood samples, any disruption in erythrocyte integrity can adversely impact the quality of the plasma, and its subsequent analysis for rare biomarker detection.^[^ ^19^, ^20^, ^21^, ^22^ ^]^ This disruption can be caused by hydrodynamic stresses applied to the cells during plasma extraction.

To assess the hydrodynamic forces being applied on the cells, and any hemolysis occurrence, the channel Reynolds number and the shear stress were calculated at three main parts of the device: at the first contraction‐expansion area (CEA), at the middle CEA, and at the last CEA. Two locations at each CEA were evaluated: the main flow streamlines of the cells at the centreline of the CEAs and at the cross‐section of the side channel at those CEAs (Figure S4, Supporting Information).

The results show that for the main flow streamlines, the shear stress has a maximum of 24 Pa for all three CEAs, and the Reynolds number has a maximum value of 489 (Figure S5 AB, Supporting Information). For the side channel cross‐sections (an area of 10 µm (width) by 20 µm (height)), the shear stress ranges from 38.2 to 105.2 Pa, and the Reynolds number has a value of maximum of 204 for all three side channels. The Reynolds numbers fall within the laminar flow regimen as expected for hydrodynamic cell focusing in microfluidics^[^ ^23^, ^24^, ^25^ ^]^ (Figure S5C, Supporting Information). The shear stress is also below the reported value (150 Pa) for RBC lysis^[^ ^26^ ^]^ (Figure S5D, Supporting Information). Experiments were done to evaluate the quality of the separated plasma with the HCHPS0.5 and compare it to the conventional plasma separation (centrifugation.) The blood plasma was separated from human whole blood (45% HCT) using both methods and the level of hemoglobin was measured (absorbance at 414 nm). The results suggest that the level of hemoglobin in the plasma separated by centrifugation is at 0.35 a.u., and this value drops by 54% for the plasma separated using HCHPS0.5 reducing to 0.16 a.u. Figure 4C). The similarly reported values,^[^ ^19^, ^22^ ^]^, promise a better quality of plasma separated with microfluidic systems, facilitating an improved downstream biomarker analysis.

To investigate the stability of the HCHPS0.5 device for separating plasma, the device was used to separate plasma continuously for 30 min with a flow rate of 15 mlh^−1^. During this operation time, every 5 min, the plasma and waste outlets were collected and analyzed for purity and yield of the plasma. The results show that the plasma purity from the device agreed with the initial experiments (Figure 4A‐i) and had consistent values of 47.5%, 45.5%, and 41.5% (average of 45%) over 15 min of operation, and there was no significant difference between the collected batches of plasma (Figure 4D‐i). However, this purity was decreased to 22.3%, 17.6%, and 10.9%, at time points 20, 25, and 30 min‐post separation, respectively, which will not be desired for a biosensing application. In the case of yield, device can operate stably with a yield of 19.6%, 19.4%, and 19.1% for a duration of 15 min (timepoints 5, 10, and 15 min, respectively), and on average with a yield of 18.9% over half an hour, which again is in agreement with the initial device characterization (Figure 4D‐ii). With this characterization, we conclude that a single HCHPS0.5 device can have a stable performance for separating plasma from about 4 ml of whole blood

### 2.4 Biomarker Recovery and Lactate Sensing

An aptamer bead‐based fluorscence biosensing and electrochemical aptamer‐based biosensing were performed to evaluate and compare the conservation of plasma quality obtained via two separation methods – one using conventional centrifugation, and second, using the microfluidic HCHPS0.5 device. Blood samples were spiked with different concentrations of lactate, a small molecule generated during anaerobic cellular respiration as a model biomarker, and then plasma samples containing lactate were derived using two different approaches (conventional centrifugation or on‐chip separation using HCHPS0.5 device) for bead‐based fluorscence biosensing detection.

**Figure 5:**
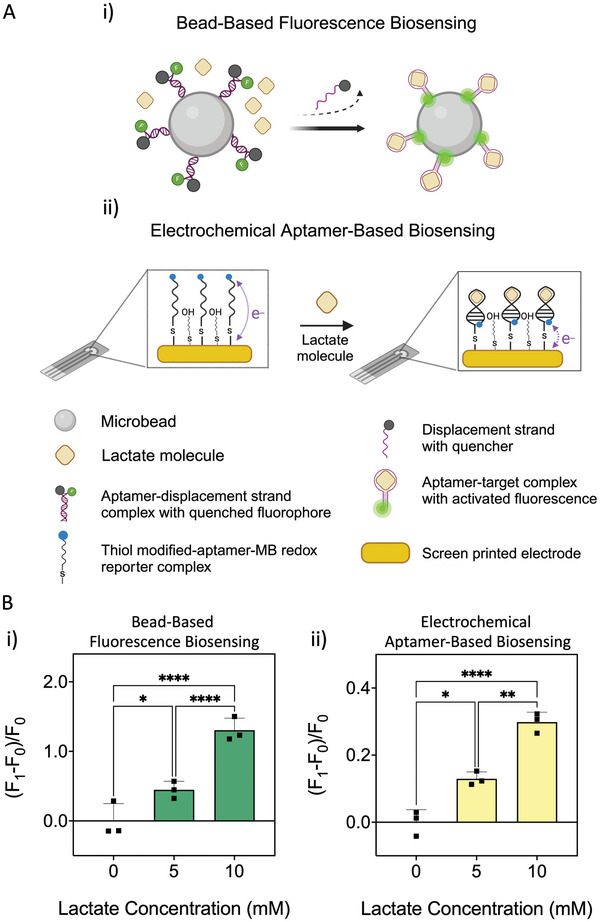
Biomarker recovery and lactate sensing. A) shows the schematic of the biosensing methods used for lactate measurments in the blood plasma. (i) shows the bead‐based fluorscence biosensing and (ii) shows the electrochemical aptamer‐based biosensing. B) The results show that on‐chip extraction produces a plasma with admissable quality, which makes sensitive detection of lactate possilbe in two different biosensing methods. F_0_ is the fluorescent signal or kinetic differential measurement (KDM%) for the lowest concentration (0 pM), and F_1_ is the fluorescent signal or kinetic differential measurement (KDM%) for for each sample with higher concentrations. The data shows the mean ± standard deviation of three replicates. The comparisons between groups (in Figure 5; Figure S3, Supporting Information) are done with two‐way ANOVA with Tukey's multiple comparisons test: 0.1234(ns), 0.0021(**), 0.0002(***), < 0.0001(****).

First, the bead‐based fluorscence biosensing described here incorporates aptamers as biorecognition probes (**Figure** **5**A‐i) where the aptamer specific to lactate was chosen from a recent work.^[^ ^27^ ^]^ Fluorescein (FAM)‐conjugated capture aptamer specific to lactate is prehybridized with a displacement strand conjugated to a quencher. The proximity of the organic fluorophore with a quencher causes its emitted fluorescence intensity to be diminished. This quenched complex is functionalized onto the beads and incubated with its target in a heterogenous biological matrix. The aptamer binds preferentially to the target which causes the removal of the displacement strand. The subsequent increase in fluorescence signal is caused by the quencher and fluorophore no longer being in proximity to one another. The increase in fluorescence intensity is therefore a function of target concentration.

Second, for the electrochemical lactate aptamer‐based sensing, the sensors were fabricated using screen‐printed electrodes with three‐electrode systems (silver/silver chloride (Ag/AgCl) reference electrode as well as gold (Au) counter and working electrodes (WE). The WE surface was functionalized with the previously reported lactate aptamer^[^ ^27^ ^]^ whose one end was conjugated with thiol and the other end was labeled with methylene blue (MB), a redox reporter. The thiol group enables aptamer immobilization on the gold surface through gold‐thiol self‐assembled chemistry.^[^ ^28^ ^]^ The MB redox reporter signals the presence of the target of interest^[^ ^29^, ^30^ ^]^ In the absence of lactate, the MB redox reporter is distanced from the electrode surface; thus, the electron transfer happens slowly, and a lower current is observed^[^ ^31^ ^]^ (Figure 5A‐ii). Once the aptamers recognize and bind lactate, they undergo a conformational change that brings the MB‐modified terminus of the aptamer closer to the surface of the electrode, leading to a faster electron transfer and producing a higher electrochemical response^[^ ^29^, ^30^, ^31^ ^]^(Figure 5A‐ii).

The difference in the electron transfer kinetic at these two unbound and bound states forms the principle of Kinetic differential measurement (KDM) and is commonly used for reporting the signals produced by EAB sensors.^[^ ^32^ ^]^ In this method, the sensor is scanned at two different frequencies; one known as the signal‐on frequency (typically has higher values) is the frequency at which the target‐bound aptamers are triggered and the produced current increases by the increase in target concentration, and the other frequency is known as signal‐off frequency (typically a lower frequency), which triggers the unbound aptamers, and the magnitude of current decreases with an increase in concentration. The KDM percentage is then calculated through the following equation:

(1) $$\begin{array}{c} KDM\%=\frac{\left(Current_{signal\;on}-Current_{signal\;off}\right)}{2(Current_{signal\;on}+Current_{signal\;off})}\times 100 \end{array}$$

The KDM method minimizes the sensor‐to‐sensor variations and prevents drift in the signal over time and in complex biofluids.^[^ ^33^, ^34^ ^]^ In this work also, we used the KDM method to report the produced signal. The blood samples that were spiked with a higher concentration of lactate showed larger signals when their respective plasma samples were tested with the lactate EAB (Figure 5B‐ii), indicating the lactate was successfully recovered when the HCHPS microfluidic device was used for plasma separation.

Square wave voltammetry (SWV) was used to capture the generated electrochemical signal and the difference in the charge transfer kinetics between the bound and unbound states of the aptamers was used to calculate the kinetic differential measurement (KDM).^[^ ^32^ ^]^ This method, previously developed for aptamer‐based biosensors, effectively reduces sensor‐to‐sensor variability, corrects signal drift, and enhances the signal‐to‐noise ratio.^[^ ^33^, ^34^ ^]^

The results from the both biosensing methods show that the plasma exctracted by the HCHPS0.5 device has the suitable quality to perform downstream analysis. Specifically, in the aptamer bead‐based fluorscence biosensing show that we can achieve a better distinction in lactate concentrations in the plasma separated with the microfluidic chip.

As shown in Figure 5B‐i compared to Figure S3‐A (Supporting Information); although the baseline levels of both plasmas are roughly the same, however, when it comes to higher concentrations, specifically, 5 and 10 mM, there is no significant difference in the signal gain level of the concentrations in the plasma separated by the centrifugation. The reason behind this can possibly be the higher level of hemolysis in the sample preparation step using the centrifuge. This effect was less in the sample prepared by the microfluidic device (Figure 4C), and as a result, the biosensing works well enough to distinguish between concentrations successfully.

## 3 Conclusion

Here, we developed the Hydrodynamic Continuous, High‐throughput Plasma Separator (HCHPS) microfluidic device, which integrates contraction‐expansion microchannels with optimized side channels to reliably and efficiently extract plasma from human whole blood samples. Three different HCHPS devices were designed and evaluated for their performance in processing human whole blood samples. After evaluations, HCHPS0.5 (with a consistent extraction rate of 0.5%) reaches a purity of 47% and a yield of 18%, which is an improvement from the original design showing its potential for subsequent biosensing applications. This device also reaches a purity of 62% and a yield of 18% with diluted blood (22.5% HCT level). The HCHPS0.25 device also attains a purity of 64% with whole blood and 96% with diluted blood. The resulting yield remains 10% in both cases.

**Tables** **1** and **2** compare the current work with similar passive hydrodynamic microfluidic devices that have been used for plasma separation with whole and diluted blood. The passive methods for plasma separation are advantageous over the active ones because of their simplicity and being independent of external forces. Particularly for point‐of‐care diagnostics or clinical settings where an operator might not be familiar with high‐tech equipment or in the case of resource‐limited settings, these devices are more accessible, user‐friendly, and affordable to operate.

**Table 1:** Comparison of active and passive microfluidic plasma separation methods with whole blood.

Reported Work	Method	Purity%	Yield%	Throughput	HCT%
Ref. [39]	Dielectrophoresis + Sedimentation	99	0.22	0.36 mlh^−1^	45
Ref. [35]	Acoustofluidic	85–95	N.R.	1.2 mlh^−1^	45
Ref. [40]	Acoustofluidic	1–98	N.R.	0.06–0.18 mlh^−1^	45
Ref. [16]	Hydrodynamic	99 and 32	5 and 30	2 mlh^−1^ and 10 mlh^−1^	45 and 33.7
Ref. [13]	Hydrodynamic	99	1‐6	30 mlh^−1^	42
Ref. [37]	Filtration + capillary	99	25	0.96 mlh^−1^	45
Ref. [38]	Hydrodynamic + crossflow filteration	97	12	2 mlh^−1^	45
This work ‐ HCHPS0.5	Hydrodynamic	47	18	15 mlh^−1^	45
This work ‐ HCHPS0.25	Hydrodynamic	64	10	15 mlh^−1^	45

**Table 2:** Comparison of passive microfluidic plasma separation methods with diluted blood.

Reported Work	Method	Yield%	Purity%	Throughput	HCT%
Ref. [16]	Hydrodynamic	30	32	10 mlh^−1^	33.7
Ref. [13]	Hydrodynamic	1–6	99	30 mlh^−1^	31
This work ‐ HCHPS0.5	Hydrodynamic	18	62	15 mlh^−1^	22.5
This work ‐ HCHPS0.25	Hydrodynamic	10	96	15 mlh^−1^	22.5

Although active methods provide high purity of the separated plasma, they bring more complexity and dependency on external infrastructure to the system. For example, for platforms depending on acoustophoretic cell separation, there is a need for devices such as a signal generator, acoustic transducers, and amplifiers. Moreover, the developed microfluidic device was placed with an ice‐water‐filled petri to prevent overheating and bubble formation within the device.^[^ ^35^ ^]^

Additionally, the performance of the active methods needs further optimization beyond the flow rate, which can make this process more complicated. In a recent work that utilizes dielectrophoresis for plasma separation, with a decrease in the flow rate from 2.5 to 3.5 µl, the purity of the extracted plasma drops by about 50%.^[^ ^36^ ^]^ While working with whole blood, the throughput of these active platforms ranges from 0.06 to 0.36 mlh^−1^, which can be a limitation where high throughput is required.

In the case of passive methods,^[^ ^13^, ^16^ ^]^ we showed that hydrodynamic devices offer a better opportunity in continuous and high‐throughput plasma separation. We optimized a previously reported design, reaching a more balanced performance regarding the purity and yield of the extracted plasma.

There are other passive techniques, such as filtration methods.^[^ ^37^, ^38^ ^]^ These platforms tend to process samples and separate plasma in batch modes since continuous feed of whole blood causes saturations in the filters, pillars, or membranes, which are used for separation and lead to device clogging. This brings longer operational time, resulting in delays in sample analysis, which can be a limitation in continuous monitoring and sensing. Overall, the advantages of hydrodynamic‐based separation make it a promising platform for continuous plasma separation, with the potential for seamless integration into biosensing devices.

The designed devices were examined to assess its plasma quality by measuring the extent of hemolysis within the device. HCHPS0.5 showed better performance compared to the gold standard centrifugation by lysing the erythrocytes to a lesser degree. This was proven by processing human whole blood containing different concentrations of lactate for its sensitive detection by two different biosensing methods: a bead‐based fluorscence biosensing and an electrochemical biosensing. The acceptable quality of the plasma separated by HCHPS0.5, with less interference from other blood components, improves the performance of the bead‐based biosensing compared to the sample prepared by centrifugation. Also, the quality of blood is suitable for an electrochemical biosensor. Tables 1 and 2 compare the current work with similar passive hydrodynamic and active methods for microfluidic devices, which have been used for plasma separation with whole and diluted blood with a similar approach.

## 4 Experimental Section

### Materials

All reagents and material were purchased from Sigma–Aldrich unless otherwise stated. Silicon wafers were obtained from UniversityWafer, Inc. The negative photoresists, SU‐8 2010 and SU‐8 3025 were purchased from Kayaku Advanced Materials, USA. Polydimethylsiloxane (PDMS), SYLGARD 184, was purchased from Dow Corning, Ellsworth Canada. All DNA products were purchased with their respective modifications from Integrated DNA Technologies, USA. Whole blood samples were purchased from BiolVT, USA. The 10 µm streptavidin‐coated magnetic beads were obtained from Spherotech, USA.

### Microfluidic Device Fabrication

The microfluidic devices were fabricated following the conventional photolithography method. Since the designs include two different heights, a two‐layer photolithography was conducted for the fabrication of the master mold. The masks were designed by CAD software (SolidWorks, Dassault Systémes). Silicon wafers were coated with negative photoresists, SU‐8 2010 for the first layer and SU‐8 3025 for the second layer. Following the master mold patterning and fabrication by MLA150 direct write UV lithography system, (Heidelberg Instruments), PDMS was cast on the master mold, and PDMS devices were fabricated. The PDMS devices were oxygen plasma treated (15W, 30sec, 10sccm, air) (Tergeo, Pie Scientific.), and were bonded to a glass cover slip. Fabricated devices were degassed overnight using a surfactant, Pluronic F108.

### CFD Simulations

The flow within the microchannels was simulated by COMSOL Multiphysics 6.2 with the Laminar flow module (SPF). The fluid flow stream inside the microchannels follows the Navier‐Stokes equation. Then, the lengths of the side microchannels were varied, until the predetermined extraction rate was achieved. The fluid entering the channel had been supposed to have the same properties as water since was presented plasma in the CFL area. The inlet flow was assumed to be a Newtonian and incompressible fluid and reached a steady state quickly once it entered the microchannel. The inlet flow rate was set to 1 mlh^−1^, and the pressure was set equal to zero at the outlets, at the inner wall of the microchannel, a no‐slip boundary condition was assumed.

### Microscopic Imaging

For CFL formation and its area measurement the experiments were run under a microscope (ECLIPSE Ti2, Nikon) and video recordings were recorded while running samples within the device. The visualizations were done under the TRITC channel (570 nm laser). The CFL area was calculated using an MS‐Office‐based VBA macro script (Copyright(c) 2014 YOUpresent Ltd.)

### Device Evaluation

For all experiments, human whole blood was used. The sample was injected to the device by a syringe pump (Fusion 200 X, Chemyx). The plasma and waste outlets were collected in microtubes for further characterization.

Purity: the device evaluation for its purity was done with the help of a flow cytometry device (NovoCyte, Agilent). The concentration of RBCs/WBCs at plasma outlet (*C*_*p*_) and the concentration of RBCs/WBCs at the inlet (*C*_*i*_) were completely analyzed by the device. The SSC‐H versus FSC‐H were plotted and gated properly for the cells.

(2) $$\begin{array}{c} purity=\frac{C_{i}-C_{p}}{C_{i}} \end{array}$$

Yield: to evaluate the yield of each device for their extracted plasma the ratio of the extracted plasma volume (from plasma outlets, port #3) (*V*_*p*_) to total blood volume (*V*_*t*_) was calculated.

(3) $$\begin{array}{c} yield=\frac{V_{p}}{V_{t}} \end{array}$$

Hemolysis: after the plasma separation, the levels of free hemoglobin in the plasma were measured by absorbance peaks at 414 nm.

### Plasma Extraction Using a Centrifuge

The plasma samples through centrifugation were collected by processing the whole blood sample by a centrifuge at 1500 rcf for 15 min at room temperature.

### Lactate Bead‐Based Fluorscence Biosensing

The sequences for the lactate aptamer and displacement strand used in these experiments were as follows: /5BiosG/i6FAMK/TTTTTTTCTCTCGACGACGAGTAGCGCG TATGAATGCTTTTCTATGGAGTCGTC and CGTCGTCGAGAG/3IABkFQ/.^[^ ^27^ ^]^

The lactate aptamer and displacement strand system here was developed to specifically detect the presence and concentration of lactate in complex biological matrices. The lactate aptamer was hybridized to its displacement strand in the absence of a target. The proximity of a quencher molecule on the displacement strand to the fluorophore on the aptamer caused the quenching of the emitted fluorescence signal. Upon addition of the target, the lactate aptamer preferentially binded to the target, which removed the displacement strand, thus mitigating the quenching effect, and causing an increase in emitted fluorescence. Therefore, emitted fluorescence was directly proportional to the concentration of target lactate in the system.

The biotin modification on the 5^*TM*^ end of the lactate aptamer was for bead functionalization of 10 µm streptavidin‐coated magnetic beads. To begin the biosensing, lactate aptamer, and displacement strand were incubated together in SELEX buffer at room temperature for 30 min at a molar ratio of 4:1 aptamer: displacement strand. This step was to ensure the effective hybridization and quenching of the emitted fluorescence signal from the lactate aptamer. SELEX buffer ensured the optimal activity of aptamer and displacement strand by maintaining pH and ion concentration. The recipe for SELEX buffer was 500 mM NaCl, 10 mM MgCl_2_, and 10 mM KCl in 50 mM pH 7.5 HEPES. Following hybridization, the complex was functionalized on streptavidin‐coated magnetic beads by incubation at room temperature for 60 min, followed by a wash step by magnetic separation. Plasma obtained from centrifugation and microfluidic device was then spiked with 0, 5 mM, or 10 mM lactate, and functionalized beads were incubated with these samples for 40 min at room temperature. Finally, beads from each sample were washed and subsequent fluorescence intensity was measured via flow cytometry. Samples were run in triplicates to obtain median fluorescence intensity and standard deviation.

### actate Electrochemical Aptamer‐Based Biosensing

To fabricate the electrochemical aptamer‐based biosensor (EAB) for lactate detection, the previously reported protocols with slight modifications were used.^[^ ^41^, ^42^, ^43^ ^]^ For this purpose, the commercially available screen printed electrodes with gold working and counter electrode and silver refrence electrode were used. To clean the electrodes prior to fabrication, they were rinsed with isopropanol, and water and blotted with a KimWipe. Then the electrodes underwent electrochemical cleaning via ten cyclic voltammetry (CV) scans with a potential range between 0 and 1.5 V at a scan rate of 0.1 Vs^−1^ and a sampling interval of 0.005 V in 0.1 M H_2_SO_4_. The electrodes were thoroughly rinsed with MilliQ water and blotted with a KimWipe. The lactate aptamer solution was also made by mixing 2 µm of the thiol‐modified aptamer with 200 µm of TCEP in the buffer and incubated (to get reduced) in the dark and ambient condition for 1 h. Then 5 µl of the reduced aptamer was drop casted on the working electrode in dark overnight. Following the aptamer deposition, a 100 mM 2‐Mercaptoethanol MCH solution was deposited on the surface of the working electrode for 10 min in the dark at room temperature. Following the last step the electrode was rinsed in buffer, dried, and stored until use. The lactate aptamer used for fabricating the lactate sensor same as the one used in the bead‐based fluorscence biosensing, and it was previously reported.^[^ ^27^ ^]^

### Detecting Lactate in Blood Samples with the EAB

To assess lactate recovery in plasma samples isolated with the HCHPS microfluidic device, first the blood samples were spiked with different concentrations of lactate, and their associated plasma was extracted via the HCHPS0.5 device. The level of lactate in the resulting plasma samples was detected with the fabricated EAB developed for lactate detection^[^ ^31^ ^]^ To detect the level of lactate in the plasma samples, 30 µl of the sample was drop casted on the surface of the fabricated electrodes and incubated for 10 min, followed by performing the square wave voltammetry (SWV) electrochemical measurement. The SWV measurements were performed in a potential range of 0 to ‐0.5 V and a scan rate of 0.1 V s ^−1^ and at the frequencies of 200 Hz (as the signal‐on frequency) and 15 Hz (as the signal‐off frequency). The sensor^*TM*^s response (KDM%) to different concentrations of lactate was calculated via the following equation:

(4) $$KDM\%=\frac{\left(Current_{signal\;on}-Current_{signal\;off}\right)}{2(Current_{signal\;on}+Current_{signal\;off})}\times 100$$

The SWV measurements were performed in a potential range of 0 to ‐0.5 V and a scan rate of 0.1 V s ^−1^.

### Statistical Analysis:

All the statistical analysis was conducted using GraphPad Prism 10. All data were demonstrated as mean ± SD of three experimental replicates. Other than Figure 3C (explained below), there was no data pre‐processing prior to data analysis. All simulation data in Figure 2 show the results for a single run. The simulation results were consistent for multiple runs, thus only a single run is shown in the graphs. In Figure 3C, the heatmap shows the formed CFL area ratio (*F*/*F*_*min*_) for one experimental replicate; CFL areas (*F*) were normalized to CFL area with whole blood at 5 mlh^−1^ (*F*_*min*_) of the HCHPS0.5 device with human whole blood. In Figure 4, all experimental data show the mean ± standard deviation of three replicates. In Figure 4A,B, for purity and yield characterizations, the comparisons between groups were done with two‐way ANOVA with Tukey's multiple comparisons test. p‐values: 0.1234(ns), 0.0021(**), 0.0002(***), <0.0001(****). In Figure 4C, for assessment of separated plasma's quality, the comparisons between groups were done with an unpaired t‐test with Welch's correction. p values: 0.001 to 0.01 (**). In Figure 4D, for device stability analysis, the comparisons had been done by ordinary one‐way ANOVA. p values: 0.1234 (ns), 0.0332 (*), 0.0021 (**), 0.0002 (***), <0.0001 (****). In Figure 5, all experimental data show the mean ± standard deviation of three replicates. In Figure 5B, for biosensing experiments, the comparisons between groups were done with two‐way ANOVA with Tukey's multiple comparisons test. p‐values: 0.1234(ns), 0.0021(**), 0.0002(***), < 0.0001(****). In Figure S3 (Supporting Information), all experimental data show the mean ± standard deviation of three replicates. In Figure S3 (Supporting Information), for biosensing experiments, the comparisons between groups were done with two‐way ANOVA with Tukey's multiple comparisons test. p‐values: 0.1234(ns), 0.0021(**), 0.0002(***), < 0.0001(****). All simulation data in Figure S5 (Supporting Information), panels A, B, C, and D, show the results for a single run. The simulation results were consistent for multiple runs; thus, only a single run was shown in the graphs.

### Ethics Approval

The human whole blood K2EDTA samples were purchased from BioIVT, ELEVATING SCIENCE®, and had been tested with FDA CBER licensed screening tests (Title 21‐CFR PART 610.40) for pathogens. All in vitro studies in this work were conducted in a biosafety Level 2 lab and were approved by IRB.

## Author Contributions

H.A., F.K., S.A.H., and M.P. conceived the initial concept. H.A. and S.A.H. developed and executed the CFD studies and designed microdevices. H.A., F.K., and S.A.H. conducted the experiments. F.K. and S.S. designed and executed the electrochemical and bead‐based biosensings. All authors wrote, edited, discussed, and approved the manuscript for submission.

## Conflict of Interest

The authors declare no conflict of interests.

## Supporting information

Supporting Information

## Data Availability

The data that support the findings of this study are available from the corresponding author upon reasonable request.
