# Supplementary material for: Continuous High‐Throughput Plasma Separation for Blood Biomarker Sensing Using a Hydrodynamic Microfluidic Device
Source: Adv Healthc Mater. 2025 Feb 19;14(9):2404193. doi: 10.1002/adhm.202404193 (PMC11973946; doi:10.1002/adhm.202404193)
Supplement: Supplementary file 1 — Supporting Information [file ADHM-14-0-s001.pdf]

ADVANCED HEALTHCARE MATERIALS

Supporting Information

for *Adv. Healthcare Mater.*, DOI 10.1002/adhm.202404193

Continuous High-Throughput Plasma Separation for Blood Biomarker Sensing Using a Hydrodynamic Microfluidic Device

*Hesam Abouali, Fatemeh Keyvani, Seied Ali Hosseini, Sanjana Srikant and Mahla Poudineh**

Supporting Information for "Continuous High-Throughput Plasma Separation for Blood Biomarker Sensing Using a Hydrodynamic Microfluidic Device"

Hesam Abouali[1], *Fatemeh Keyvani*[1], *Seied Ali Hosseini*[1], *Sanjana Srikant*[1], *Mahla Poudineh*[*,1]

[1]Department of Electrical and Computer Engineering, University of Waterloo
Waterloo, Ontario, N2L 3G1, Canada
Email Address: mahla.poudineh@uwaterloo.ca

Cell-free layer formation

To evaluate the HCHPS designs for their ability to form cell-free layers (CFL), each design was tested with whole blood at different flow rates of 5, 10, and 15 ml/hr. The higher flow rates helped with better formation of CFL.

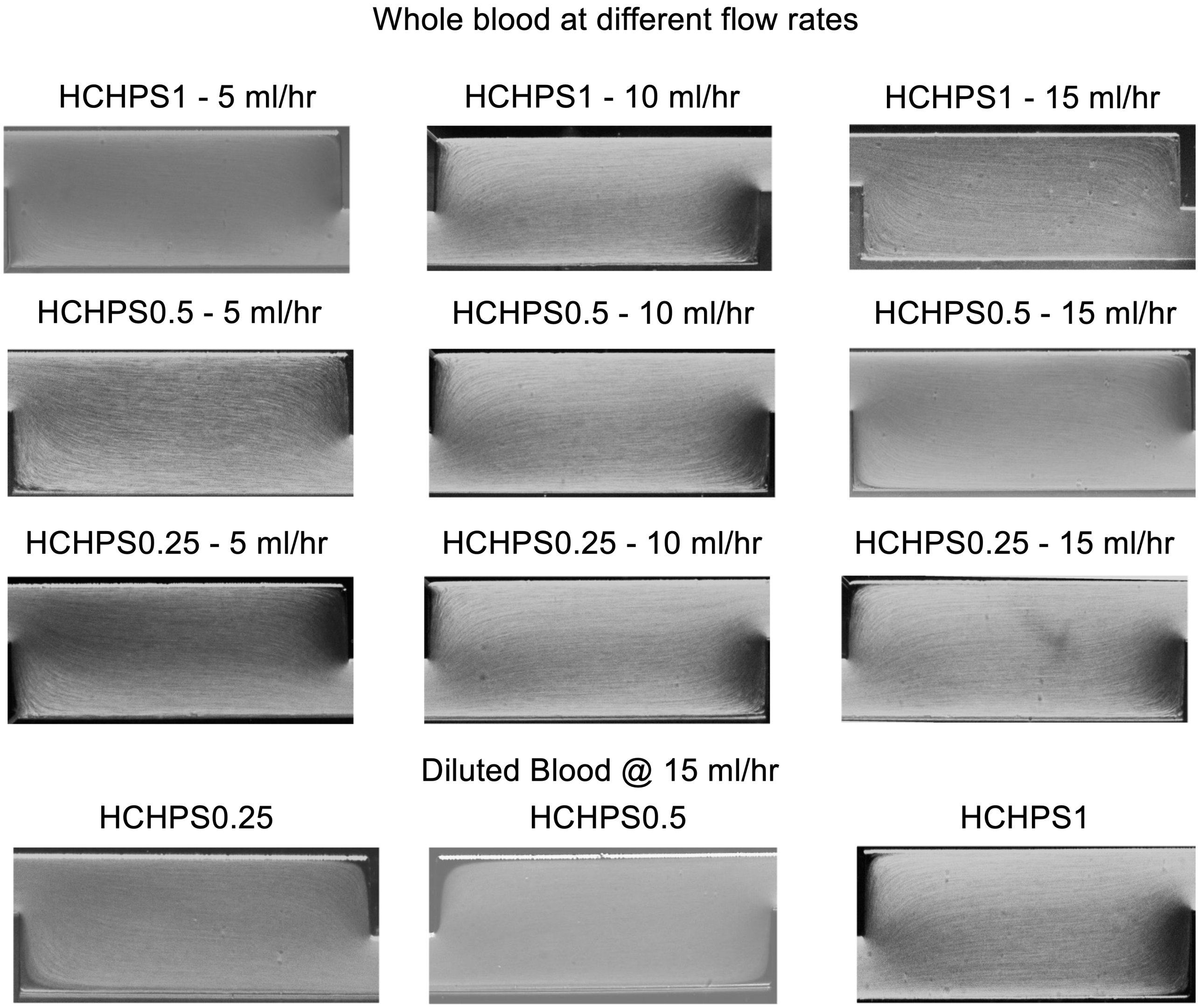

Figure S1: CFL formation. CFL was captured under the microsscope for its formation under different conditions for various HCHPS devices. The HCHPS0.5 device shows larger CFL formed compared to HCHPS1, and HCHPS0.25 has larger CFL compared to HCHPS0.5. However, the yield of the separated plasma will be in the opposite order.

Flow Cytometry Sample Data

To evaluate the performance of the device regarding its outlets were collected and were analyzed by volume for its yield, and the number of RBCs/WBCs for the purity. The flow cytometry data (SSC-H vs. FSC-H, Gate R1) are used to study the absolute count of the cells in plasma and waste/blood cell outlets.

Here we show an example of one replicate of HCHPS0.5 device working at the flow rate of 10 ml/hr with whole blood with a yield of 18.71% and a purity of 11.05%.

The collected volume for plasma outlet side #1, plasma outlet side #2, and the blood waste outlets were 85.4µl, 117.2µl, and 880.8µl respectively. With the results shown in Figure S2A (abs. count or i.e. concentration [cells/ml]), we can calculate the number of cells at each outlet. These numbers will be 10.38×10^6, 7.37×10^6, and 88.84×10^6 cells in plasma#1, plasma#2, and waste outlets respectively.

Thus, the total number of cells at the inlet will be 1.066×10^9 cells (the sum of all cells at all outlets) in a total injected volume of 1083.4 µl of blood entering the device (the sum of all volumes at all outlets) which results in a concentration of 0.984×10^9 cells/ml.

The average plasma outlet concentration of 0.875×10^9 cells/ml from this replicate, we will have the following for purity of this replicate:

$$yield = \frac{volume \quad of \quad plasma \quad outlets}{total \quad volume \quad of \quad plasma \quad and \quad blood \quad outlets} \tag{1}$$

$$yield\% = \frac{85.4\mu l \quad + 117.2\mu l}{85.4\mu l + 117.2\mu l + 880.8\mu l} \times 100 = 18.71\% \tag{2}$$

$$purity = \frac{Cells \quad concentrations \quad at \quad the \quad inlet - Cells \quad concentrations \quad at \quad the \quad plasma \quad outlet}{Cells \quad concentrations \quad at \quad the \quad inlet} \tag{3}$$

$$purity\% = \frac{0.984 \times 10^9 - 0.875 \times 10^9}{0.984 \times 10^9} \times 100 = 11.05\% \tag{4}$$

A)

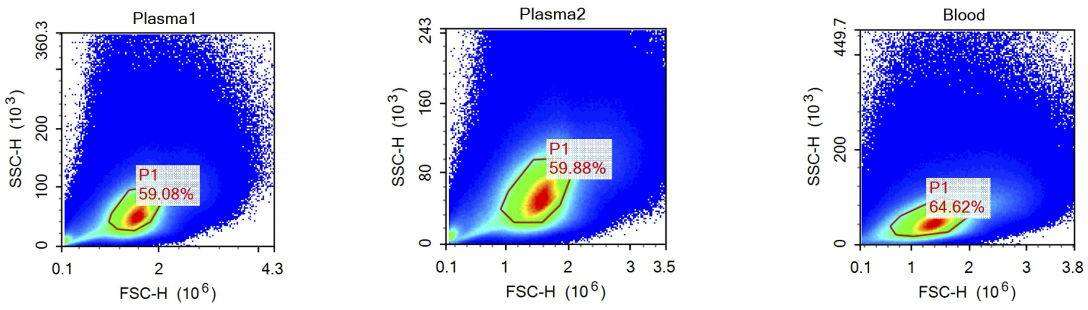

Sample Statistics of Plasma1

Gate	Count	Abs. Count
All	5,573,040	
P1	3,292,597	864,141,200

Sample Statistics of Plasma2

Gate	Count	Abs. Count
All	5,608,216	
P1	3,358,380	886,389,200

Sample Statistics of Blood

Gate	Count	Abs. Count
All	5,121,198	
P1	3,309,411	1,008,658,400

Figure S2: HCHPS0.5 device purity and yiled analysis. The outlets from the device were collected and analyzed to calculate the yield and the purity. The flow cytometry data (abs. count) show the concentration [# cells/ml] of the RBCs/WBCs at each outlet/inlet.

Lactate assays for plasma separated by centrifugation

The bead-based fluorscent and electrochemical assays were also done for the plasma separated by centrifugation. The results from the bead-based assay shows a possible interference from higher hemolysis in the plasma prepared by the centrifugation whih makes the distinction between 5 and 10 mM concentrations infeasible in this assay.

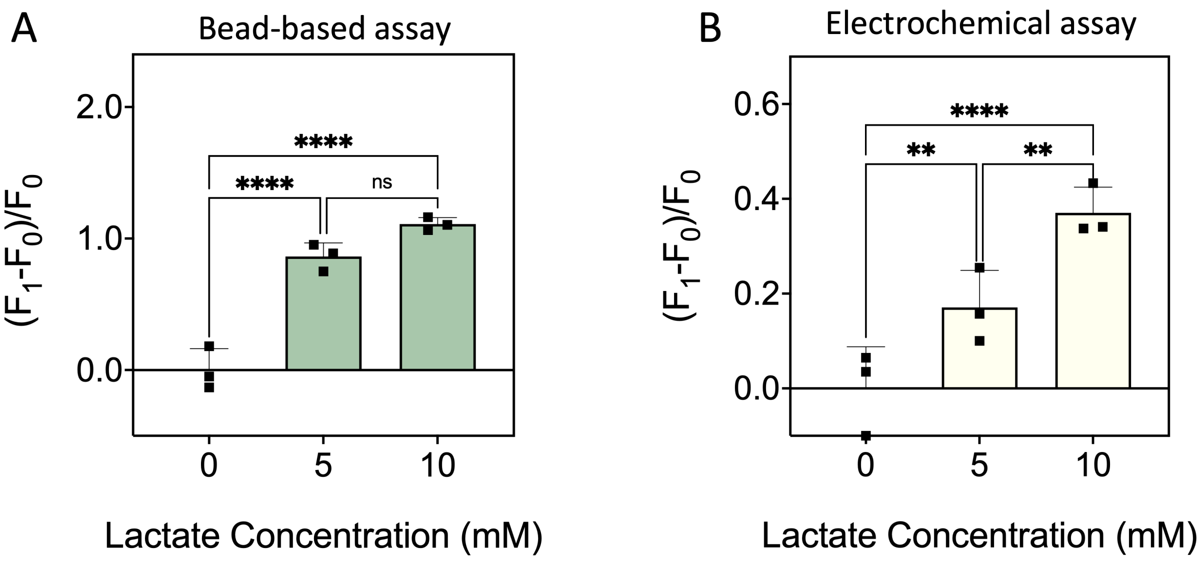

Figure S3: Lactate assays results for plasma generated by centrifugation. F_0 is the fluorescent signal orkinetic differential measurement (KDM%) for the lowest concentration (0 pM), and F_1 is the fluorescent signal or kinetic differential measurement (KDM%) for for each sample with higher concentrations. The data shows the mean ± standard deviation of three replicates. The comparisons between groups (in FigureS3 and Figure 5) are done with two-way ANOVA with Tukey's multiple comparisons test: 0.1234(ns), 0.0021(**), 0.0002(***), < 0.0001(****).

CFD studies for hydrodynamic forces

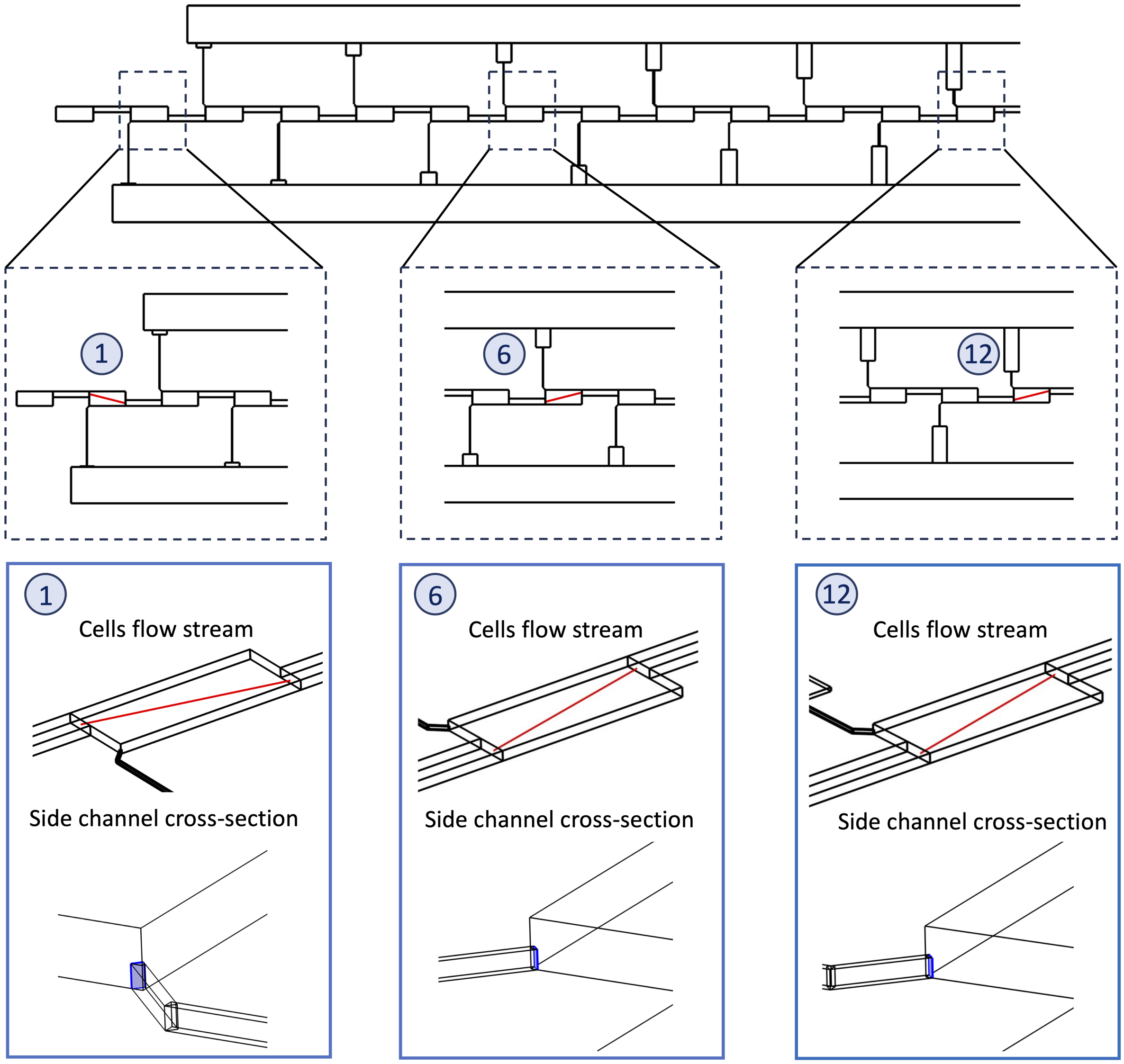

Figure S4: eometries for CFD evaluations of hydrodynamic criteria. The shear stress and the Reynolds number were evaluated for the cell streamline which is located at the centre of a CEA. The streamline at the 1st, 6th, and 12th CEAs were evaluated. Also, the cross-sections of side channels at the same CEAs were measured for the shear stress and the Reynolds number.

CFD studies for hydrodynamic forces

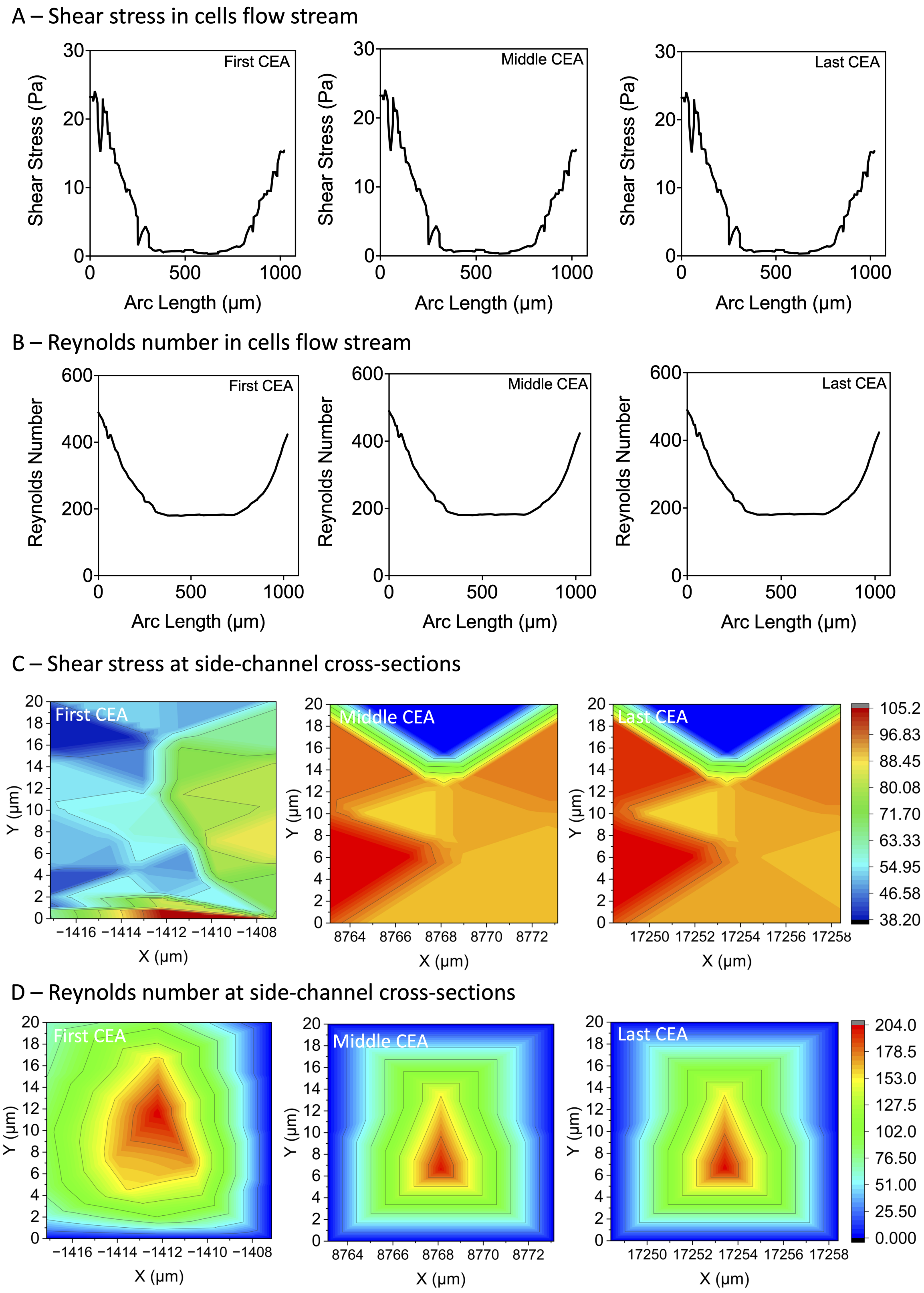

Figure S5: CFD simulation of shear stress and Reynolds number. A) shear stress was evaluated for the cell streamline at the centre of 1st, 6th, and 12th CEAs, which is comparable at all three CEAs, and reaches a maximum of 24 Pa. B) The Reynolds number reaches a maximum value of 489. C) the shear stress was also measured for the cross-section of side channels of the same CEAs, where it reaches 105 Pa at the 1st CEA side channel. D) for the same side channels the Reynolds numbers were calculated. Each data point shows the result from a single simulation. The simulation results were consistent for multiple runs, thus only a single run is shown in the graphs.
